# Trastuzumab upregulates programmed death ligand-1 expression through interaction with NK cells in gastric cancer

**DOI:** 10.1038/s41416-020-01138-3

**Published:** 2020-10-26

**Authors:** Kohei Yamashita, Masaaki Iwatsuki, Noriko Yasuda-Yoshihara, Takeshi Morinaga, Yosuke Nakao, Kazuto Harada, Kojiro Eto, Junji Kurashige, Yukiharu Hiyoshi, Takatsugu Ishimoto, Yohei Nagai, Shiro Iwagami, Yoshifumi Baba, Yuji Miyamoto, Naoya Yoshida, Jaffer A. Ajani, Hideo Baba

**Affiliations:** 1 Department of Gastroenterological Surgery, Graduate School of Medical Sciences, Kumamoto University, Kumamoto, Japan; 2 Department of Gastrointestinal Medical Oncology, The University of Texas MD Anderson Cancer Center, Houston, TX 77030 USA

**Keywords:** Gastric cancer, Cancer immunotherapy

## Abstract

**Background:**

The predictive significance of programmed death ligand 1 (PD-L1) for programmed death 1 (PD-1) inhibitors remains unclear in gastric cancer (GC) due to the dynamic alteration by treatments. We aimed to elucidate the effects of trastuzumab (Tmab) on PD-L1 expression in GC.

**Methods:**

PD-L1 expression was evaluated by multicolour flow cytometry analysis after co-culturing GG cell lines and immune cells with Tmab. IFN-γ in the co-culture experiments was quantified. Immunohistochemistry (IHC) for PD-L1 expression using clinical samples was also performed to confirm PD-L1 alteration by Tmab.

**Results:**

PD-L1 expression was significantly upregulated by Tmab in *HER2*-amplified GC cell lines co-cultured with peripheral blood mononuclear cells (PBMCs). PD-L1 upregulation by Tmab was also observed in the GC cells co-cultured with NK cells in time-dependent manner, but not with monocytes. IFN-γ concentration in conditioned media from co-cultured PBMCs and NK cells with Tmab was significantly higher and anti-IFN-γ significantly suppress the Tmab-induced PD-L1 upregulation. IHC also suggested PD-L1 upregulation after Tmab treatment.

**Conclusions:**

Tmab can upregulate PD-L1 expression on GC cells through interaction with NK cells. These results suggest clinical implications in the assessment of the predictive significance of PD-L1 expression for PD-1 inhibitors.

## Background

Gastric cancer (GC) is the fifth most common type of cancer and the third leading cause of cancer death worldwide.^1^ Despite recent progress in multidisciplinary treatments, prognosis for patients with advanced GC remains poor. Cancer immune therapy, including immune checkpoint inhibitors (ICIs), is highlighted as a new paradigm against cancer via interaction with host immune systems.^2^ Programmed death 1 (PD-1) is an immune checkpoint molecule that is expressed on several types of immune cells, including effector T cells. It induces exhaustion of the effector function and apoptosis of the immune cells by engaging its ligand, programmed death ligand 1 (PD-L1). As some cancer cells can escape from host immune surveillance by utilising this pathway,^3,4^ ICIs that target the PD1/PD-L1 axis are considered a breakthrough therapy for several cancer types, including GC.^5,6^

Identifying predictive biomarkers for PD-1/PD-L1 blockade can help stratify patients and maximise therapeutic benefits. Several predictive markers, such as tumour mutation burden, microsatellite instability, and tumour-infiltrating lymphocytes status, have been reported.^7^ Among them, tumour PD-L1 expression is considered a logical biomarker because PD-L1 is a substantial target of the ICIs. However, patients with PD-L^−^ GC tumours also benefited in clinical trials of efficacy of PD-1 inhibitors.^8,9^ The predictive value of PD-L1 expression for PD-1/PD-L1 blockade is, therefore, controversial.

A possible reason for this discrepancy is inaccurate assessment of PD-L1 expression when dynamic changes in PD-L1 expression during treatment are not considered. Although several studies have shown some cytotoxic agents and radiotherapy to induce PD-L1 upregulation in several cancer types,^10–13^ previous clinical trials have not addressed the influence of previous treatment on PD-L1 status. Therefore, whether such changes can occur in GC should be confirmed. However, few studies have examined it so far, and none have addressed the effects of molecular-target agents on changes in PD-L1 expression in GC.

Trastuzumab (Tmab) is a humanised monoclonal antibody against the extracellular domain of human epidermal growth factor receptor-2 (HER2). Tmab treatment became the golden standard for *HER2*-amplified GC after its efficacy was shown in a large-scale phase III trial (the ToGA trial).^14^ Potential mechanisms of Tmab’s anti-tumour activity include inhibition of intracellular signal-transduction pathways by binding HER2, induction of HER2 internalisation, and activation of antibody-dependent cell-mediated cytotoxicity (ADCC) by immune effector cells, mostly natural killer cells (NK cells) and monocytes.^15–17^ Emerging evidence also associates Tmab with both a direct cytotoxic effect through the release of perforin and granzyme B, and an adaptive immune response by secreting inflammatory cytokines, including interferon-γ (IFN-γ).^18,19^ These findings suggest that Tmab upregulates PD-L1, as IFN-γ is known to upregulate PD-L1.^20^

In this study, we hypothesised that Tmab can upregulate PD-L1 expression in GC by interacting with immune cells. To elucidate the effect of Tmab on changes in PD-L1 expression, we examined PD-L1 expression after co-culturing *HER2*-amplified GG cell lines and peripheral blood mononuclear cells (PBMCs). We also isolated NK cells and monocytes from PBMCs, and performed similar co-culture assays to determine the contribution of these cells to changes in PD-L1 expression after Tmab treatment. Then, we validated the alteration of PD-L1 expression using clinical samples with immunohistochemistry (IHC).

## Methods

### Cell lines and cell culture

NCI-N87 and NUGC4 cells, which are *HER2*-amplified GC cell lines^21,22^ were selected for the following experiments using Tmab. Both cell lines were obtained from the Japanese Collection of Research Bioresources Cell Bank and Riken BioResource Center Cell Bank. The selected GC cell lines were grown in RPMI 1640 medium (FUJIFILM Wako Pure Chemical Co., Osaka, JP) supplemented with 10% inactivated foetal bovine serum (FBS; Sigma-Aldrich, St. Louis, MO, USA), and were incubated at 37 °C in a humidified chamber containing 5% CO_2_.

### Co-culture assay of GC cell lines and PBMCs

PBMCs were isolated from whole human blood from healthy donors (*n* = 3) by a density gradient centrifugation using Ficoll-Pague PLUS (GE Healthcare Life Sciences, Little Chalfont, UK) with manufacturer’s protocols. The selected GC cell lines were treated with Tmab (Chugai Pharmaceutical Co., Tokyo, JP) and human immunoglobulin G (hIgG) (rituximab; Chugai Pharmaceutical Co., Tokyo, JP) at a concentration of 5 μg/ml, in mono-culture or direct co-culture assays with the isolated PBMCs at a ratio of 1:4 (GC cell lines: PBMCs) for 48 h. In addition, the same direct co-culture assays were performed at various co-culture intervals of 6, 12, 24 and 48 h to observe temporal alterations of PD-L1 expression. After the Tmab treatment, the conditioned media were collected and preserved at −80 °C until their next use in enzyme-linked immunosorbent assays (ELISA). Culture plates were washed with phosphate-buffered saline (PBS; Wako), and cells were harvested by incubation with Accutase reagent (Innovative Cell Technologies, Inc., San Diego, CA, USA) at 37 °C until cells detached. Harvested cells were used for subsequent multicolour flow cytometry.

### Suppression of HER2 on GC cell lines by synthetic small-interfering RNAs

HER2 on the selected GC cell lines were transiently downregulated using two predesigned Silencer Select siRNA directed against ERBB2 (ERBB2 siRNAs human s611; #1 and s613; #2, Thermo Fisher Scientific) and a non-targeting siRNA (Invitrogen, Thermo Fisher Scientific) was used as a negative control. 24 h after plating, the cells were transfected with the HER2-siRNAs or control siRNA using Lipofectamine RNAiMAX (Invitrogen, Thermo Fisher Scientific) according to the manufacturer’s protocol. After transfection for 24 h for NCI-N87 and 72 hours for NUGC4, the GC cells were harvested and used for subsequent co-culture assay of PBMCs with Rmab, Tmab, and an antibody to Fc gamma receptor (#564220, eBioscience, Thermo Fisher Scientific, at a concentration of 5 μg/ml).

### Isolation of NK cells and monocytes, and co-culture with GC cell lines

NK cells and monocytes were isolated from PBMCs of healthy donors (*n* = 3) by immunomagnetic negative selection, using the EasySep Human NK Cell Enrichment Kit and the EasySep Human Monocytes Enrichment Kit (Stemcell Technologies Inc., Vancouver, BC, CA) by their manufacturer’s protocols. The purity of NK cells and monocytes in the isolated cells was then confirmed as ≥90% by multicolour flow cytometry.

The selected *HER2*-amplified GC cell lines and NK cells were directly and indirectly co-cultured at a ratio of 4:1 (GC cell lines: NK cells; based on the general proportion of NK cells to PBMCs) with Tmab or hIgG in the same manner as the co-culture assays with PBMCs. To maintain NK cell viability and activity, KBM 502 (Kohjin Bio Co., Saitama, JP) which is a medium that contains human IL-2, was used for the co-culture assays. Similarly, the selected HER2-amplified GC cell lines and monocytes were co-cultured at a ratio of 3:2 (GC cell lines: monocytes; also based on the proportion of monocytes in PBMCs) in RPMI 1640 medium with 10% inactivated FBS, with or without Tmab. After the treatment, the conditioned media and co-cultured cells were collected in the same way as the co-cultured assay with PBMCs, and used for subsequent experiments.

### Multicolour flow cytometry analysis

Multicolour flow cytometry staining was performed immediately after the cultured cells were harvested. Single-cell suspensions (0.5–1 × 10^6^ cells) were prepared and pre-incubated with Fc-receptor blocking solution (Biolegend, San Diego, CA, USA) for 10 min at room temperature to reduce non-specific binding. The single-cell suspensions were then stained with fluorochrome-conjugated anti-human antibodies and isotype-matched antibodies for 30 min at 4 °C, based on two multicolour panels. Details of the two multicolour panels are shown in Supplementary Table 1. In brief, the lymphocyte surface panel was designed to confirm the purity of NK cells and monocytes. It contained fluorochrome-conjugated anti-human antibodies against CD14, CD66b, CD3, CD4, CD8, CD45, CD56 and CD19 (BioLegend). The tumour cell-surface panel was designed to distinguish tumour cells from lymphocytes, and to examine cell-surface markers, including PD-L1, on tumour cells. It contains fluorochrome-conjugated anti-human antibodies against CD340 (HER2), CD274 (PD-L1), CD45 and CD326 (EpCAM) (BioLegend). After staining, excess antibodies were washed away twice with PBS and single-cell suspensions were stained with 7-amino actinomycin D (7-AAD; Beckman Coulter, Brea, CA, USA) for 20 min at room temperature to detect apoptotic cells. Flow cytometry was performed on a BD FACSVerse instrument (BD Biosciences, Franklin Lakes, NJ, USA); data were analysed on FlowJo 10 software (Tree Star, Ashland, OR, US) and represented by histograms and mean fluorescence intensity (MFI).

### Gating strategies in multicolour flow cytometry

To confirm the purity of NK cells and monocytes after isolation from PBMCs, we performed gating using the lymphocyte surface panel. After gating to single cells, CD45^+^ cells were gated to discriminate white cells from GC. Among the cell population, NK cells were detected as CD3^−^/CD56^+^ cells and monocytes as CD14^+^ cells. Gating strategies for NK cells and monocytes are shown in Supplementary Figs. 1, 2.

To analyse live GC cells after Tmab treatment, we performed gating with the tumour cell-surface panel. After gating to single cells, EpCAM^+^ cells were gated to discriminate GC cells. Among them, 7-AAD^−^ cells were further gated; this cell population was used for later analysis of live GC cells after Tmab treatment. This gating strategy for live GC cells is shown in Supplementary Fig. 3.

### Intracellular flow cytometry analysis

Intracellular flow cytometry analysis was performed to evaluate alterations of intracellular PD-L1 expression after co-culture of the HER2-amplified GC cell lines and PBMCs with Tmab or hIgG. After the cultured cells were harvested, single-cell suspensions (0.5–1 × 10^6^ cells) were prepared and stained with Fixable Viability Stain 620 (BD Biosciences) for 10 min at room temperature to discriminate viable cells from dead cells. After pre-incubation with Fc-receptor blocking solution, cell surface markers of CD45 and EpCaM were stained with fluorochrome-conjugated anti-human antibodies. Fixation and permeabilisation were then performed with Stabilizing Fixative (BD Biosciences) and saponin-based permeabilisation and wash reagent (Thermo Fisher Scientific, Waltham, MA, USA). Finally, intracellular staining with fluorochrome-conjugated anti-human antibodies against CD274 (PD-L1) was performed and flow cytometry analysis was conducted.

### NK cells activation markers and NK cells killing assay

The HER2-amplified GC cell lines (NCI-N87 and NUGC4) and NK cells were co-cultured at a ratio of 4:1 with Tmab or hIgG for 3 h. The co-cultured cells were then harvested and performed flow cytometry analysis of NK cells activation markers, including CD69 (APC/Cy7-conjugated antibody) and CD107a (PE/Cy7-conjugated antibody).

NK cells killing against GC was monitored using an IncuCyte Live cell Analysis System (Essen BioScience, Tokyo, Japan) by manufacturer’s protocols of immune cell killing assay. In brief, target GC cells labelled with CytoLight Rapid Red Reagents (Sartorius, Tokyo, Japan) and NK cells were seeded into the 96-well plate at a ratio of 4:1 (GC cell lines: NK cells) with RPMI 1640 or KBM 502 medium. Then, Annexin V Green Reagent (Sartorius) and Tmab or hIgG at a concentration of 5 μg/ml were added. Finally, the apoptotic GC cells counts were acquired by the IncuCyte Live cell Analysis System with image acquisition every 3 h. The data were triplicated and represented over time.

### Quantification of IFN-γ by ELISA

IFN-γ in conditioned media from the co-culture experiments was quantified using Human IFN-γ Quantikine ELISA Kit (R&D Systems) by manufacturer’s protocols. In brief, properly diluted conditioned media were added to wells coated with polyclonal antibody specific for IFN-γ, and incubated for 2 h at room temperature, followed by additional sequential incubations with polyclonal antibody against IFN-γ conjugated to horseradish peroxidase for 2 hours. Following a wash to remove any unbound antibody-enzyme reagent, a substrate solution is added to the wells. After the colour developed, absorbance was measured at 450 nm for optical density using a microplate reader. IFN-γ concentration in each well was then calculated using a standard curve.

### Neutralisation of IFN-γ in co-culture assay of HER2-amplified GC cell lines and NK cells

A neutralising antibody to IFN-γ (clone NIB42, eBioscience) at concentration of 10 μg/ml was used to neutralise IFN-γ in co-culture assay of the HER2-amplified GC cell lines and NK cells with Tmab. After the co-culture for 48 h, PD-L1 expression on the HER2-amplified GC cell lines were measured by multicolour flow cytometry in the same manner as previous experiments.

### Immunohistochemical staining

Paired samples before and after Tmab treatment were obtained from patients with GC in our institute (*N* = 9). Formalin-fixed paraffin-embedded tissue samples were sectioned at 5 μm. After deparaffinisation, heat-induced antigen retrieval was performed in a steamer autoclave at 121 °C for 15 min with antigen retrieval solution (for PD-L1 and pan-cytokeratin AE1/AE3, pH 9, Histofine; Nichirei Biosciences, Tokyo, Japan; for CD45 and NKp46, pH6, Dako REAL Target Retrieval Solution; Dako Japan, Tokyo, Japan). Endogenous peroxidase activity was blocked using 3% hydrogen peroxide. Slides were then incubated with monoclonal primary antibodies against PD-L1 (clone E1L3N, 1:200 dilution; Cell Signaling Technology, Danvers, MA, USA), pan-cytokeratin AE1/AE3 (ab27988, 1:100 dilution; Abcam, Cambridge, UK), CD45 (D9M8I, 1:200 dilution; Cell Signaling Technology), and NKp46 (clone 195314, 1:100 dilution; R&D Systems, Minneapolis, MN, US) overnight at 4 °C. Slides were then incubated with anti-rabbit or anti-mouse EnVision™+/horseradish peroxidase secondary antibody (Dako Japan) and visualised with 3,3-diaminobenzidine. Counterstained with haematoxylin was performed and evaluated by experienced pathologists who were unaware of the clinical data. PD-L1 expression on GC cells was evaluated as percentage of PD-L1 stained cancer cells and NK cell infiltration was evaluated by the presence of NKp46 positive cells within the tumour.

### Statistical analysis

Each experiment was performed in triplicate. Representative data for consistent results are shown. The data are presented as mean values with standard deviation. A two-tailed unpaired Student’s *t* test was used to compare two groups. All statistical analysis was performed using JMP version 13.1 software (SAS Institute, Cary, NC, USA). *P* < 0.05 was considered significant.

## Results

### PD-L1 expression is upregulated by Tmab in HER2-amplified GC cells co-cultured with PBMCs

**Fig. 1:**
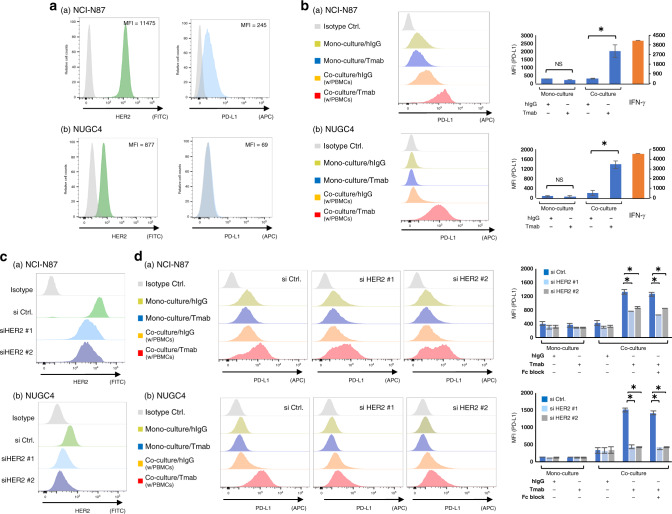
PD-L1 upregulation by Tmab in *HER2*-amplified GC cells co-cultured with PBMCs. **a** HER2 and PD-L1 expression on *HER2*-amplified GC cell lines (NCI-N87 and NUGC4). **b** PD-L1 expression on the GC cell lines that were monocultured or co-cultured with PBMCs, and treated with Tmab or hIgG (5 μg/ml) for 48 h. PD-L1 expression on the GC cell lines treated with a recombinant IFN-γ (10 ng/mL) were displayed as a positive control. **c** Suppression of HER2 on the GC cell lines by small-interfering RNAs. **d** PD-L1 expression on the *HER2*-suppressed GC cell lines that were monocultured or co-cultured with PBMCs, and treated with Tmab or hIgG. HER2 and PD-L1 expression were measured by multicolour flow cytometry. The data are represented by histograms and MFI values. NS, no significant difference. *P < 0.05.

HER2 and PD-L1 expression on the *HER2*-amplified GC cell lines (NCI-N87 and NUGC4) were measured by flow cytometry analysis (Fig. 1a). Among the selected GC cell lines, NCI-N87 displayed higher HER2 expression (MFI = 11475), followed by NUGC4 (MFI = 877). We next performed co-culture assay of the *HER2*-amplified GC cell lines and PBMCs with Tmab. Tmab did not alter PD-L1 expression on the monocultured GC cell lines, but significantly increased PD-L1 expression on those co-cultured with PBMCs (NCI-N87, added hIgG MFI = 308, added Tmab MFI = 2003, *P* < 0.05; NUGC4, added hIgG MFI = 195, added Tmab MFI = 1380, *P* < 0.05) (Fig. 1b). To examine the role of HER2 expression for the observed Tmab-induced PD-L1 upregulation, we silenced HER2 on the selected GC cell lines by si RNA (Fig. 1c) and performed the same co-culture assay with Fc block. As shown in Fig. 1d, suppression of HER2 and Fc block significantly decrease the PD-L1 upregulation by Tmab (NCI-N87, si HER2 #1 without Fc block MFI = 756 and with Fc block MFI = 659, si HER2 #2 without Fc block MFI = 869 and with Fc block MFI = 839; NUGC4, si HER2 #1 without Fc block MFI = 423 and with Fc block MFI = 413, si HER2 #2 without Fc block MFI = 426 and with Fc block MFI = 426) compared with si controls (NCI-N87, without Fc block MFI = 1324 and with Fc block MFI = 1254; NUGC4, without Fc block MFI = 1507 and with Fc block MFI = 1407) (*P* < 0.05).

### Tmab- induced intracellular PD-L1 upregulation and cell surface PD-L1 upregulation in HER2-amplified GC cells co-cultured with PBMCs in time-dependent manner

**Fig. 2:**
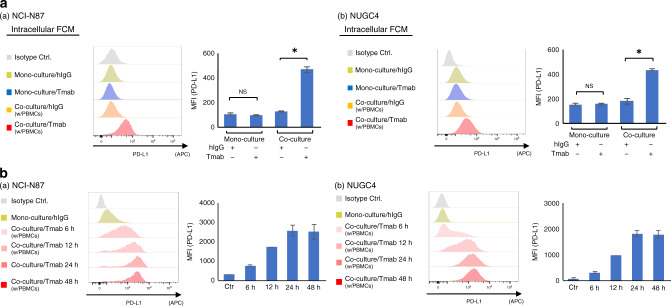
Tmab-induced intracellular and cell surface PD-L1 upregulation in *HER2-*amplified GC cells co-cultured with PBMCs in time-dependent manner. **a** Intracellular PD-L1 expression on *HER2*-amplified GC cell lines (NCI-N87 and NUGC4) that were monocultured or co-cultured with PBMCs, and treated with Tmab or hIgG (5 μg/ml) for 48 hs. **b** PD-L1 expression on the GC cell lines co-cultured with PBMCs, and treated with Tmab for 6, 12, 24 and 48 h. PD-L1 expression were measured by multicolour flow cytometry. The data are represented by histograms and MFI values.

We next examined whether Tmab can induce intracellular PD-L1 upregulation as a result of increased PD-L1 mRNA transcripts. With intracellular flow cytometry analysis, intracellular PD-L1 upregulation by Tmab was observed in both NCI-N87 and NUGC4 co-cultured with PBMCs (NCI-N87, added hIgG MFI = 125, added Tmab MFI = 467, *P* < 0.05; NUGC4, added hIgG MFI = 178, added Tmab MFI = 433, *P* < 0.05) (Fig. 2a).

Next, we performed the same co-culture assay at various co-culture intervals of 6, 12, 24 and 48 h to evaluate the time aspect of the Tmab-induced PD-L1 upregulation. As shown in Fig. 2b, Tmab upregulated PD-L1 expression on both NCI-N87 and NUGC4 co-cultured with PBMCs in time-dependent manner.

### PD-L1 expression is upregulated by Tmab in HER2-amplified GC cells directly co-cultured with NK cells

**Fig. 3:**
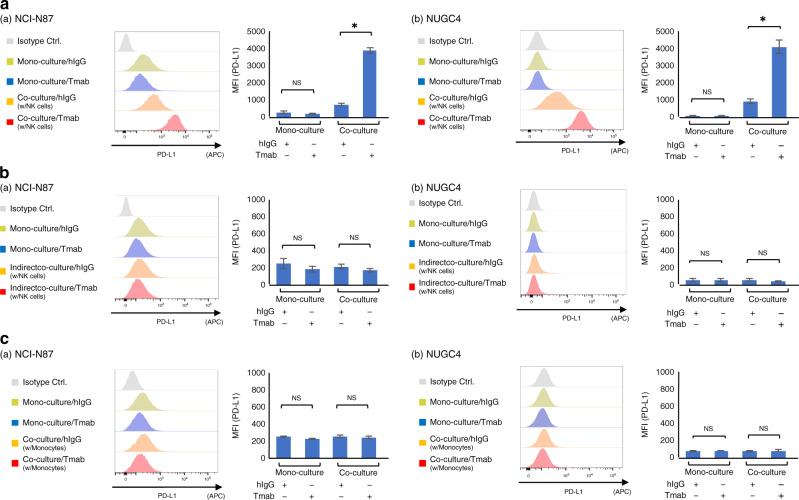
PD-L1 expression is upregulated by Tmab in *HER2*-amplified GC cells directly co-cultured with NK cells. **a** PD-L1 expression on *HER2*-amplified GC cell lines (NCI-N87 and NUGC4) that were monocultured or co-cultured with NK cells, and treated with Tmab or hIgG (5 μg/ml) for 48 h. **b** PD-L1 expression on the GC cell lines that were monocultured or indirect co-cultured with NK cells, and treated with Tmab or hIgG. **c** PD-L1 expression on the GC cell lines that were monocultured or co-cultured with monocytes, and treated with Tmab or hIgG. PD-L1 expression were measured by multicolour flow cytometry. The data are represented by histograms and MFI values. NS, no significant difference. *P < 0.05.

To identify the type of effector immune cells involved in PD-L1 upregulation by Tmab, we co-cultured GC cells with each type of effector immune cell. As previous reports indicated that NK cells and monocytes display higher ADCC while T cells and B cells were ineffective in Tmab-induced killing against cancer cell,^16,17^ we examined roles of NK cells and monocytes as a main regulator of this PD-L1 upregulation. At first, we isolated NK cells from PBMCs and co-cultured them with the *HER2*-amplified GC cell lines in the same way as we had co-cultured them with PBMCs. Consequently, PD-L1 expression on both GC cell lines was upregulated in co-culture with NK cells compared with monocultured cells. This upregulation was significantly enhanced by Tmab in both GC cell lines that were co-cultured with NK cells (NCI-N87, added hIgG MFI = 705, added Tmab MFI = 3892, *P* < 0.05; NUGC4, added hIgG MFI = 885, added Tmab MFI = 4092, *P* < 0.05) (Fig. 3a). Moreover, we demonstrated that Tmab did not alter PD-L1 expression on the *HER2*-amplified GC cell lines co-cultured with NK cells in indirect co-culture assay (Fig. 3b), suggesting direct interaction between GC cells and NK cells are required for the PD-L1 upregulation.

To examine the role of monocytes in PD-L1 upregulation, we next isolated monocytes from PBMCs and co-cultured them with GC cells, with or without Tmab. In contrast to the co-culture assay with NK cells, PD-L1 did not upregulate in GC cells co-cultured with monocytes, even after Tmab exposure (Fig. 3c).

### Tmab-induced PD-L1 upregulation is correlated with NK cell activation

**Fig. 4:**
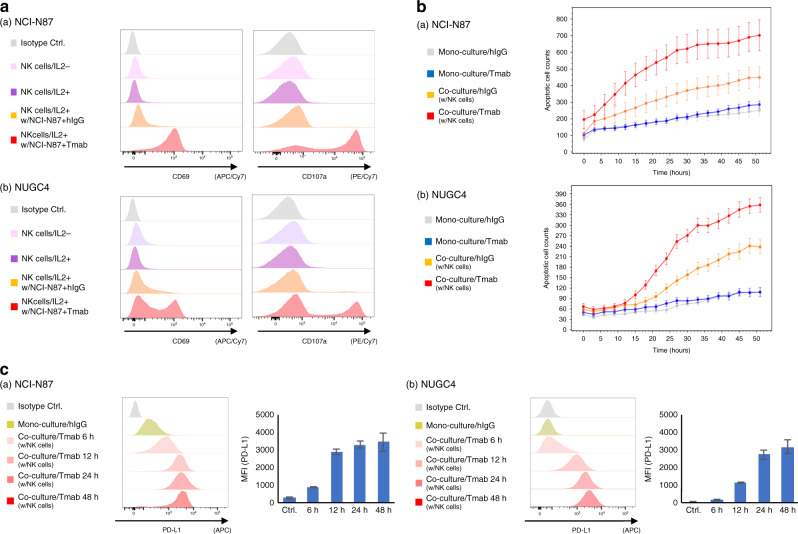
Tmab-induced PD-L1 upregulation is corrlated with NK cell activation. **a** CD69 and CD107a expression on NK cells that were monocultured or co-cultured with *HER2*-amplified GC cells (NCI-N87 and NUGC4), and treated with Tmab or hIgG (5 μg/ml) for 3 hours measured by multicolour flow cytometry. The data are represented by histograms and MFI values. **b** The apoptotic *HER2*-amplified GC cells (NCI-N87 and NUGC4) GC cells counts in monoculture or co-culture with NK cells, and treated with Tmab or hIgG (5 μg/ml). The represented data were acquired by the IncuCyte Live cell Analysis System with image acquisition every 3 h. The data were triplicated and represented over time. **c**. PD-L1 expression on the GC cell lines co-cultured with NK cells, and treated with Tmab (5 μg/ml) for 6, 12, 24 and 48 h. The data are represented by histograms and MFI values.

To confirm the NK cell activation by Tmab, flow cytometry analysis regarding NK cell activation was performed. Among NK cells co-cultured with the HER2-amplified GC cells, subpopulations with higher activation markers of NK cells, such as CD69 and CD107a, were emerged after Tmab treatment (Fig. 4a). Consequently, Tmab enhanced the killing ability of NK cells against GC cells through the induction of cell apoptosis in time-dependent manner (Fig. 4b). As with the cells co-cultured with PBMCs, we next performed the similar co-culture assay of GC cells and NK cells at various co-culture intervals of 6, 12, 24 and 48 h to evaluate the time aspect of the Tmab-induced PD-L1 upregulation. As a result, Tmab upregulated PD-L1 expression in live GC cells co-cultured with NK cells in time-dependent manner (Fig. 4c). Taken together, it was suggested that Tmab enhances anti-tumour effect via induction of NK cell activation, but conversely upregulates PD-L1 expression in surviving GC cells after Tmab treatment.

### IFN-γ is associated with PD-L1 upregulation after Tmab treatment

**Fig. 5:**
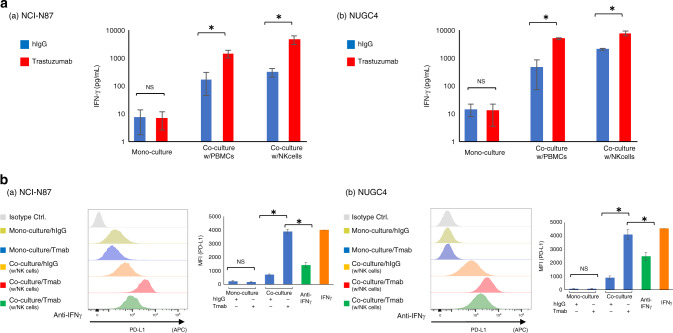
IFN-γ is associated with Tmab-induced PD-L1 upregulation in *HER2-*amplified GC cells co-cultured with NK cells. **a** IFN-γ concentrations in conditioned media from co-culture experiments of *HER2*-amplified GC cells (NCI-N87 and NUGC4) with PBMC and NK cells by ELISA. **b** PD-L1 expression on *HER2*-amplified GC cells (NCI-N87 and NUGC4) that were monocultured or co-cultured with NK cells, and treated with Tmab or hIgG (5 μg/ml) plus a neutralising IFN-γ antibody (10 μg/ml) for 48 h by multicolour flow cytometry. The data are represented by histograms and MFI values. NS, no significant difference. *P < 0.05.

In considering the mechanism of PD-L1 upregulation by Tmab thorough the interaction with immune cells, especially NK cells, we supposed cytokines secreted by these activated immune cells would affect the upregulation. IFN-γ is secreted by activated NK cells, and is known to increase PD-L1 expression as an extinct regulator. Thus, we used ELISA to examine IFN-γ concentrations in the conditioned media from the co-culture experiments of PBMC and NK cells. We found that the IFN-γ concentrations in conditioned media from co-culture experiments with PBMC and NK cells with Tmab was significantly higher than those with control hIgG (*P* < 0.05; Fig. 5a). Moreover, a neutralising antibody to IFN-γ significantly decreased the Tmab-induced PD-L1 upregulation in the GC cells co-cultured with NK cells (*P* < 0.05; Fig. 5b). Therefore, it suggests that Tmab upregulates PD-L1 by increasing IFN-γ secretion from activated NK cells.

### PD-L1 expression can be altered in clinical samples after Tmab treatment

**Fig. 6:**
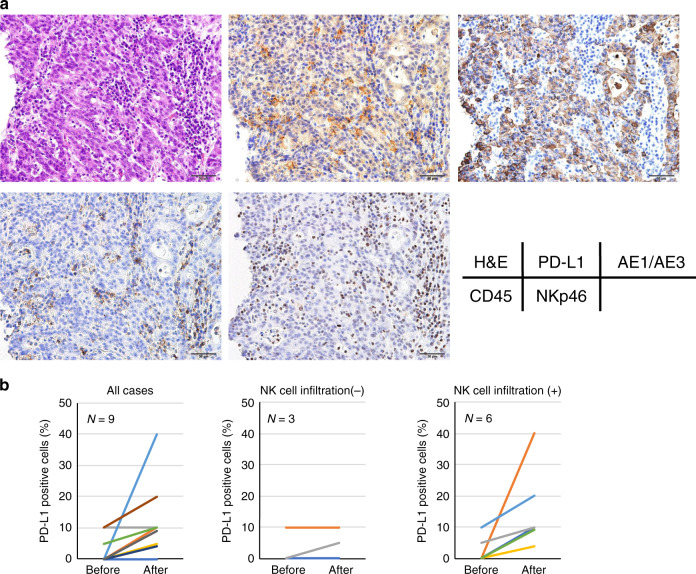
PD-L1 expression can be altered in clinical samples after Tmab treatment. **a** Representative images of H&E and IHC staining for PD-L1, pan-cytokeratin AE1/AE3, CD45, and NKp46 in tissue sample from patients with GC who received Tmab treatment. The original magnification of the image is ×200. **b** Comparison of PD-L1 expression in tissue samples before and after Tmab treatment by IHC analysis.

Finally, we performed immunohistochemistry (IHC) staining for PD-L1 and NKp46, which is one of the cell surface markers of NK cells, using tissue samples from the patients with GC who received Tmab treatment. As PD-L1 is also expressed not only on GC cells but on some types of immune cells including lymphocytes, we also performed IHC staining for pan-cytokeratin AE1/AE3 and CD45 to distinguish GC cells from immune cells. We identified that NK cells infiltrated near tumour cells and the tumour cells and immune cells expressed PD-L1 in cases after Tmab treatment (Fig. 6a). Although it was evaluated in a limited case, PD-L1 positive cells tended to increase after Tmab treatment, especially in high NK cell infiltration cases (Fig. 6b).

## Discussion

In the current study, we demonstrated that Tmab can upregulate PD-L1 in *HER2*-amplified GC cells by interacting with immune cells. We also found that NK cells, not monocytes, are mainly involved in this upregulation, as effector immune cells. This study is the first to show dynamic alteration of PD-L1 expression on GC tumour cells by molecular-target agents. Our findings indicate that the effects of previous treatments on PD-L1 expression should be considered in assessing the significance of PD-L1 expression as a predictive biomarker for PD-1 inhibitor efficacy in GC.

PD-L1 expression is reportedly upregulated by conventional radiotherapies and cytotoxic anti-neoplastics in several cancer types,^23^ though no reports demonstrate it in GC. Such upregulation has been associated with several signal pathways,^24,25^ which implies that its cytotoxic effect directly affects PD-L1 regulation in cancer cells. In contrast, the effects of molecular-target agents on the PD-L1 regulation has not been widely studied. Chaganty et al. reported that Tmab upregulated PD-L1 expression through engagement of immune effector cells and stimulation of IFN-γ secretion in breast cancer,^26^ which is consistent with our results. However, in previous reports, conditioned media from co-culture of HER2-overexpressing breast cancer cells and PBMCs with Tmab were exposed to new breast cancer cells cultured in other plates. In the current study, we directly examined changes in PD-L1 expression on GC cells exposed to Tmab, using multicolour flow cytometry to assess the direct effect of Tmab. Thus, our approach more accurately evaluated the direct effect of Tmab on PD-L1 upregulation. Moreover, this Tmab-induced PD-L1 upregulation was decreased by suppression of HER2 on GC cells and blocking of Fc gamma receptor, suggesting this PD-L1 upregulation is associated with an interaction of HER2 on GC cells, Tmab, and Fc gamma receptor on immune cells. As Tmab is used only for patients with HER2-positive GC, our study reflects actual situation in clinical setting.

Chaganty et al. also associated PBMCs with PD-L1 upregulation by Tmab in breast cancer.^26^ However, which immune effector cells among PBMCs contribute to PD-L1 upregulation was unclear. In the current study, we examined this question using cell isolation techniques, which is suitable for subsequent culture assays. Consequently, we found that NK cells, but not monocytes, mainly affect PD-L1 upregulation by Tmab in GC, through secretion of IFN-γ. At the same time, we also demonstrated Tmab upregulates NK cell activation markers, such as CD69 and CD107a, which is consistent with previous reports.^27^ Accordingly, Tmab enhanced the killing ability of NK cells against GC cells in time-dependent manner. Taken together, our findings suggest that activated NK cells play a major role in the anti-tumour effect of Tmab, while paradoxically increasing PD-L1 expression in tumour cells by IFN-γ. Although IFN-γ plays an important role in cancer immune microenvironment, it also reportedly induces PD-L1 upregulation in several types of cancer.^28^ We firstly demonstrated those roles in GC treated by Tmab and our findings should have considerable clinical implications for accurate assessment of PD-L1 expression after Tmab treatment.

Another novelty in this study is that some GC cells, which were live even after the Tmab treatment, were shown to upregulate PD-L1. In this light, combining Tmab and PD-1 inhibitors might have an improved anti-tumour effect on HER2^+^ GC. In fact, pembrolizumab+Tmab showed clinical benefit in patients with advanced Tmab-resistant, HER2^+^/PD-L1^+^ breast cancer.^29^ As for GC patients, phase 2 clinical trials of combination therapy of pembrolizumab with Tmab, capecitabine and oxaliplatin for HER2^+^ metastatic esophagogastric adenocarcinoma are ongoing and have reported an improved anti-tumour effect (NCT02954536). We believe this study will motivate researchers and clinicians for further study and contribute to develop the combination therapies.

This study has several limitations. First, this study includes a small number of clinical samples. However, confirming these current findings using a large scale of clinical samples is challenging, because re-biopsy is not routinely performed after treatment in clinical setting. Therefore, prospective experiments or in vivo assays are necessary to verify our findings. Second, we did not assess whether PD-L1 upregulation affects the prediction of PD-1 inhibitor efficacy. An accurate assessment strategy of PD-L1 expression is needed, including re-biopsy, as we described in the evaluation of HER2 status.^30^ We believe that reconsidering the PD-L1 expression as a predictor for PD-1 inhibitor efficacy in GC could be helpful.

In conclusion, we demonstrated that Tmab can upregulate PD-L1 expression on GC cells by interacting with NK cells. Our results suggest that effects on PD-L1 expression by previous treatments should be considered in assessing the significance of PD-L1 expression as a predictive biomarker for PD-1 inhibitors.

## Supplementary information

Supplementary figure and table

## Data Availability

Individual-level data may be shared on request if all legal and ethical requirements are met. Requests should be sent to the corresponding author.
