# Supplementary material for: Trastuzumab upregulates programmed death ligand-1 expression through interaction with NK cells in gastric cancer
Source: Br J Cancer. 2020 Oct 26;124(3):595–603. doi: 10.1038/s41416-020-01138-3 (PMC7851117; doi:10.1038/s41416-020-01138-3)
Supplement: Supplementary file 1 — Supplementary figure and table [file 41416_2020_1138_MOESM1_ESM.pdf]

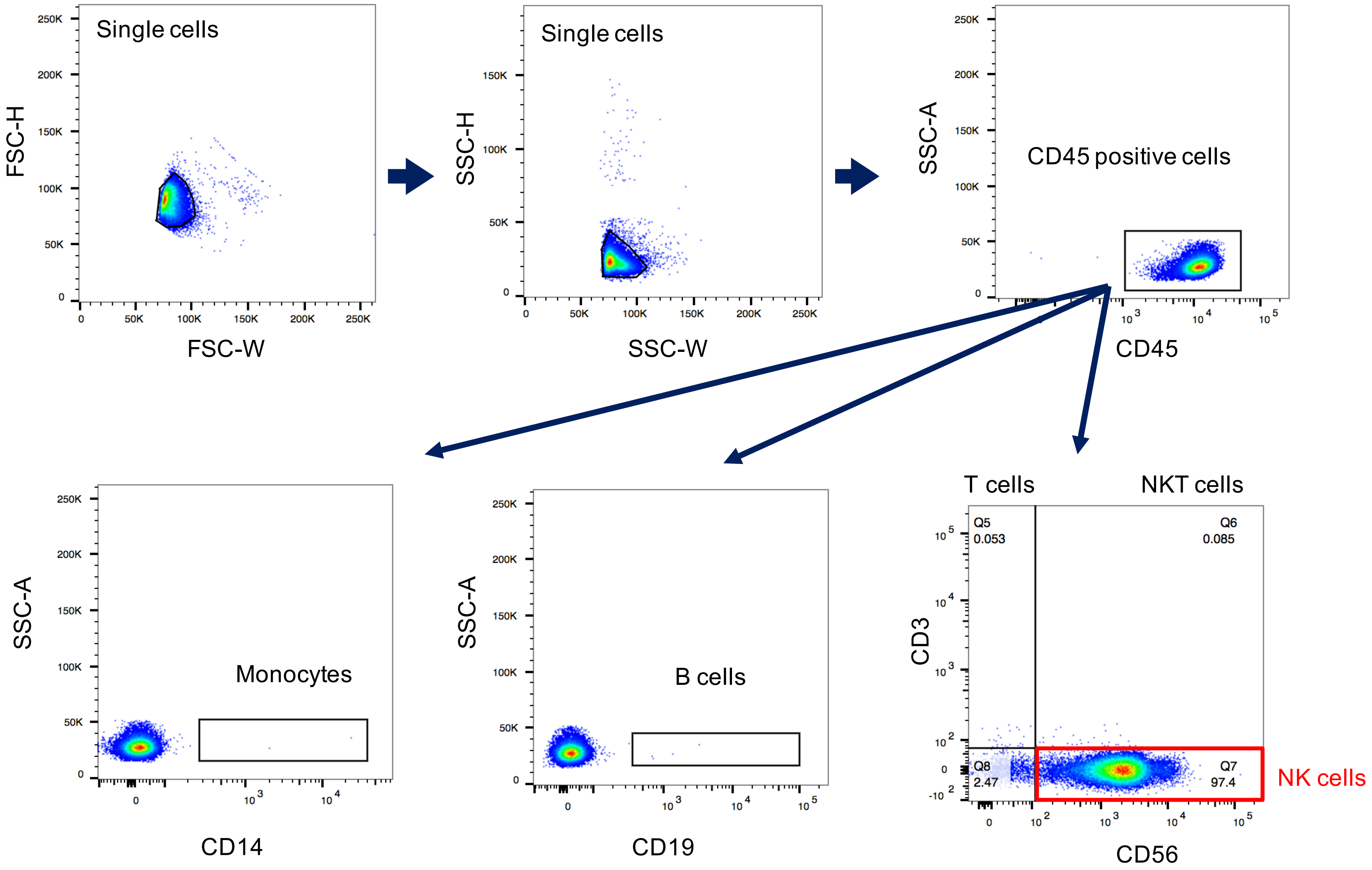

Figure S1. Gating strategy to confirm the purity of NK cells after isolation from PBMCs. After gating to single cells and CD45+ cells, NK cells were detected as CD3−/CD56+ cells. The purity of NK cells was >90 % after isolation from PBMCs.

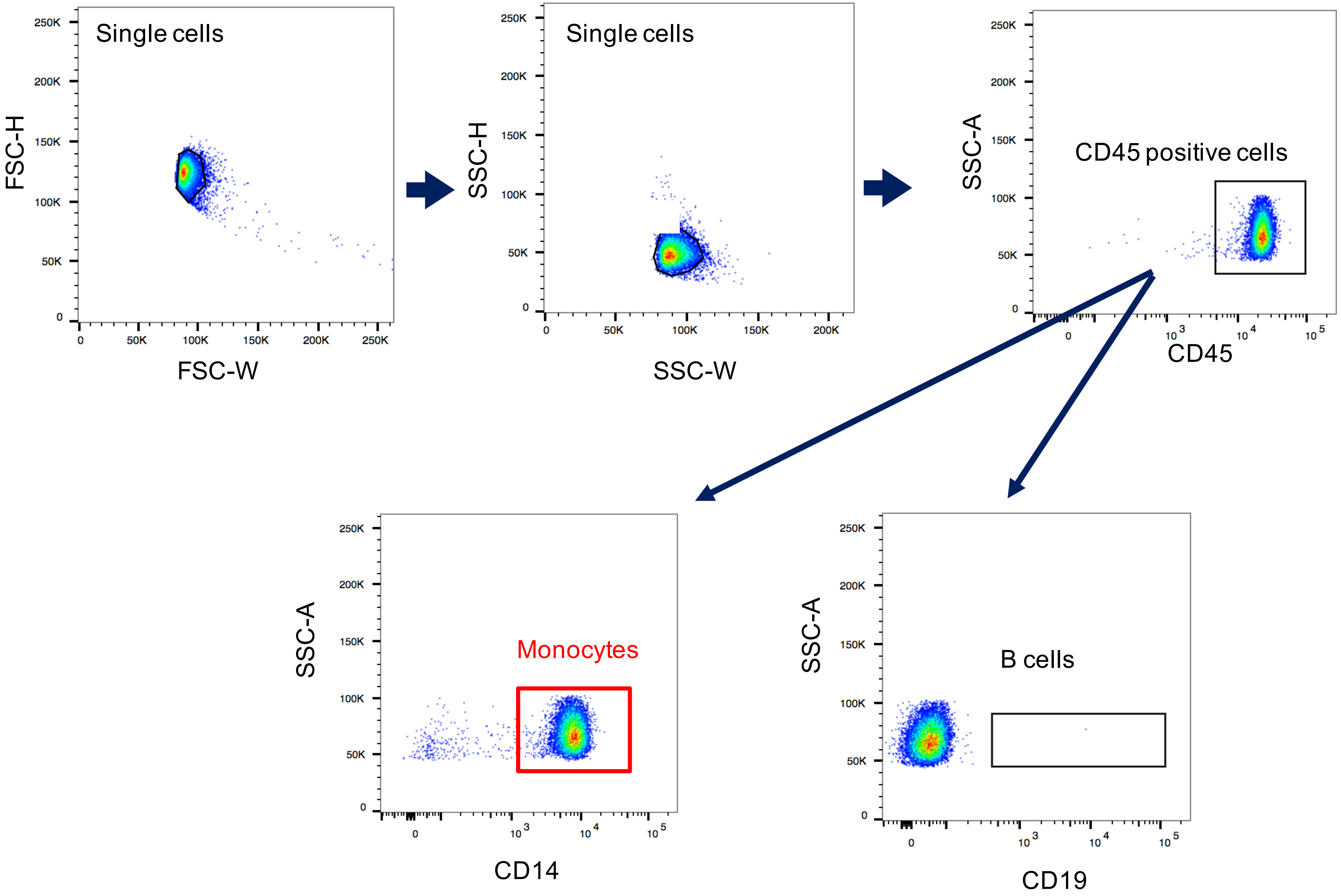

Figure S2. Gating strategy to confirm the purity of monocytes after isolation from PBMCs. After gating to single cells and CD45+ cells, monocytes were detected as CD14+ cells. The purity of monocytes was >90 % after isolation from PBMCs.

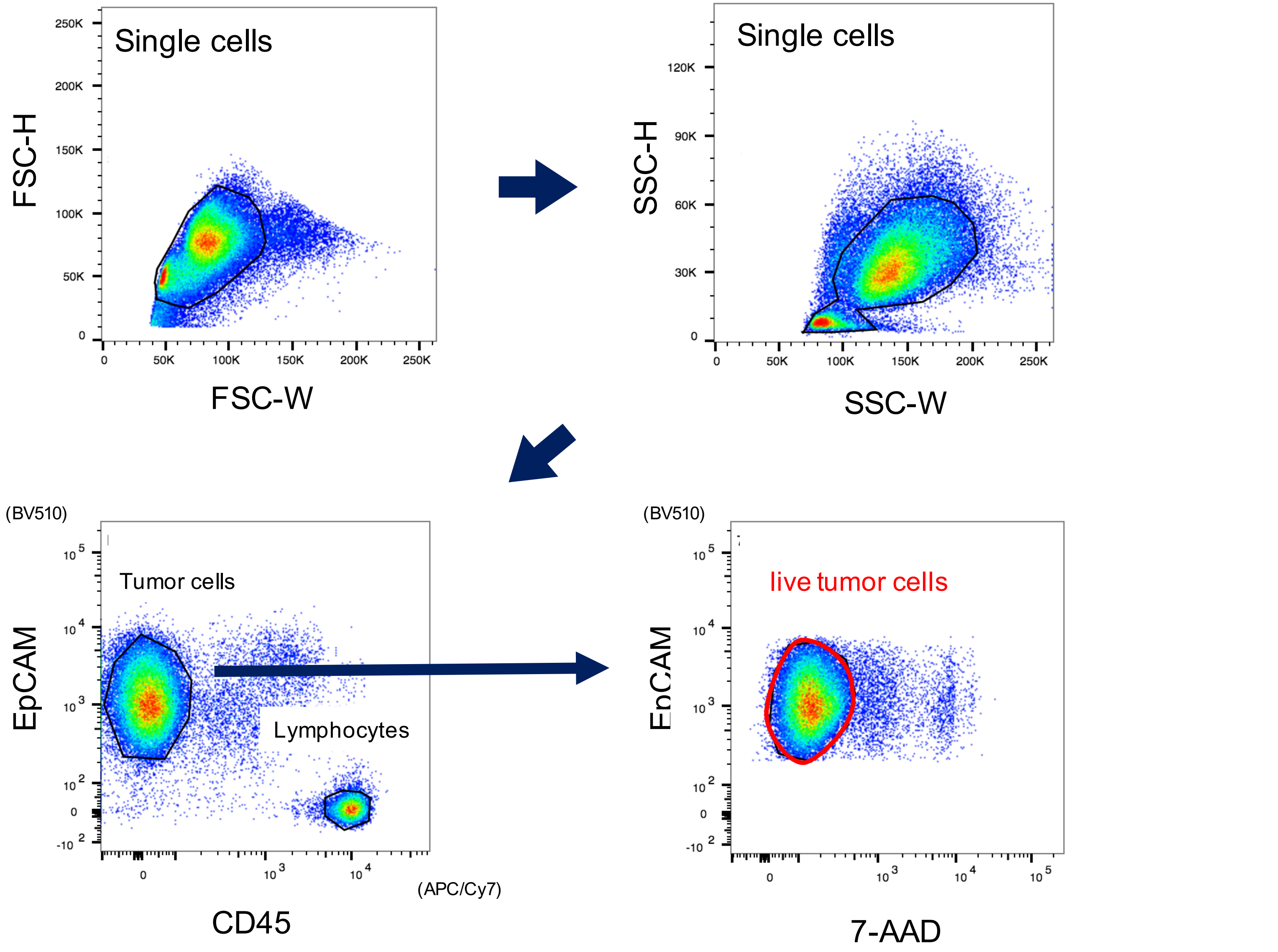

Figure S3. Gating strategy to discriminate live GC cells co-cultured with immune cells after Tmab treatment. After gating to single cells, $EpCAM^+$ cells were gated to discriminate GC cells. The $7\text{-}AAD^-$ cells were then further gated to discriminate live GC.

Table S1. Multicolor panels for multicolor flow cytometry analysis

1. Lymphocyte surface panel

	Cell surface marker	Cat.	Isotype control	Cat.
FITC	CD14	325603	M IgG1, κ	400109
PE	CD66b	305106	M IgM, κ	401609
PerCP/Cy5.5	CD3	300429	M IgG1, κ	400149
PE/Cy7	CD4	344611	M IgG1, κ	400125
APC	CD8	344721	M IgG1, κ	400121
APC/Cy7	CD45	304014	M IgG1, κ	400127
BV421	CD56	318327	M IgG1, κ	400157
BV510	CD19	302241	M IgG1, κ	400171

2. Tumor cell surface panel

	Cell surface marker	Cat.	Isotype control	Cat.
FITC	CD340 (erb/HER-2)	324404	M IgG1, κ	400109
APC	CD274 (PD-L1)	393610	M IgG1, κ	400121
APC/Cy7	CD45	368515	CD45	368515
BV510	EpCAM	324235	EpCAM	324235
